# Donor Characteristics Associated With Graft Loss and Delayed Graft Function in Very-Aged Kidney Donors: An Observational Multicentric Study

**DOI:** 10.3389/ti.2025.14862

**Published:** 2025-11-18

**Authors:** Mehdi Maanaoui, Rémi Lenain, Vivien Petit, Amaury Dujardin, Emmanuel Morelon, Xavier Charmetant, Moglie Le Quintrec, Jean Emmanuel Serre, Marc Ladrière, Sophie Girerd, Christophe Masset, Antoine Sicard, Clément Gosset, Aghilès Hamroun, Clarisse Kerleau, Claire Garandeau, François Provôt, Magali Giral, Marc Hazzan, the DIVAT Consortium, Alice Aarnink, Nicolas Abdo, Laetitia Albano, Asma Alla, Damien Ambrosetti, Laetitia Anquetil, Lionel Badet, Nadia Ben Hassen, Gilles Blancho, Mathilde Blois, Julien Branchereau, Fanny Buron, Diego Cantarovich, Agnès Chapelet, Xavier Charmetant, Luc Chauvelot, Ricardo Codas, Marion Cremoni, Jacques Dantal, Sameh Daoud, Sylvie Delmas, Clément Deltombe, Valérie Dubois, Matthieu Durand, Pascal Eschwege, Lucile Figueres, Luc Frimat, Claire Garandeau, Magali Giral, Sophie Girerd, Patricia Goldis, Clément Gosset, Caroline Gourraud-Vercel, Manon Guézennec, Jacques Hubert, Christine Kandel, Fatimaezzahra Karimi, Clarisse Kerleau, Alice Koenig, Raphaël Kormann, Marc Ladriere, François Lagrange, Emmanuelle Laurain, Pierre Lecoanet, Jean-Louis Lemelle, Moglie Le Quintrec, Thibault Letellier, Anne-Claire Lukaszewicz, Anthony Mannuguerra, Christophe Masset, Charles Mazeaud, Aurélie Meurette, Anne Moreau, Emmanuel Morelon, Vincent Pernin, Hélène Perrochia, Michael Peres, Maud Rabeyrin, Karine Renaudin, Jean-Emmanuel Serre, Antoine Sicard, Ilan Szwarc, Olivier Thaunat, Giorgo Toni, Simon Ville, Alexandre Walencik

**Affiliations:** 1 University of Lille, CHU Lille, Nephrology Department, Lille, France; 2 University of Lille, INSERM U1190, Translational Research for Diabetes, Lille, France; 3 Nantes Université, CHU Nantes, Inserm, Centre de Recherche en Transplantation et Immunologie, UMR 1064, Institut de Transplantation Urologie Néphrologie (ITUN), Nantes, France; 4 Service de Néphrologie et Immunologie Clinique, CHU Nantes, Nantes Université, ITUN, Nantes, France; 5 Service de Transplantation, Néphrologie et Immunologie Clinique, Hôpital Edouard Herriot, Hospices Civils de Lyon, Lyon, France; 6 Université Claude Bernard Lyon 1, Villeurbanne, France; 7 Department of Nephrology, Dialysis and Transplantation, Montpellier University Hospital, Montpellier, France; 8 Renal Transplantation Department, Brabois University Hospital, Nancy, France; 9 Nephrology, Dialysis and Transplantation Department, Pasteur 2 Hospital, Nice University Hospital, Nice, France; 10 Service de Santé Publique, Epidémiologie, Economie de la Santé Et Prévention, UMR1167 RID-AGE, Institut Pasteur de Lille, Inserm, CHU de Lille, Lille, France

**Keywords:** aging, elderly, kidney transplantation, donor, preservation

## Abstract

This study explores the impact of using kidneys from very-aged donors to address the organ shortage, focusing on risk factors for graft loss and delayed graft function (DGF), independent of recipient factors. Data were sourced from the French multicentric prospective DIVAT cohort and retrospectively analyzed. The study included adult recipients transplanted between 2007 and 2018 receiving kidneys from brain-deceased donors over 70. The primary endpoint was death-censored graft survival, and secondary endpoint DGF. Among 1036 patients with a median follow-up of 3.96 years (2.01–6.31), donor hypertension (HR 1.46 95% CI (1.09–1.95), cold ischemia time (HR 1.03 per hour 95% CI (1.01–1.06) and HLA mismatches (after adjustment on DGF, HR 1.98 (1.45–2.71)) were significant risk factors for graft loss. Considering DGF, donor serum creatinine (HR 1.01 95% CI (1.01–1.01) per μmol/L), warm and cold ischemia times (HR 1.01 95% CI (1.0–1.01) per minute and HR 1.05 95% CI (1.02–1.08) per hour) and the use of SCOT preservation solution (HR 3.90 95% CI (1.26–11.84)) were deleterious, while hypothermic perfusion machine was protective (HR 0.65 95% CI (0.43–0.99)). The findings emphasize the paucity of modifiable variables associated with long-term outcomes in very-aged donors and the need for peri-transplant preservation strategies.

## Graphical Abstract

**Figure:**
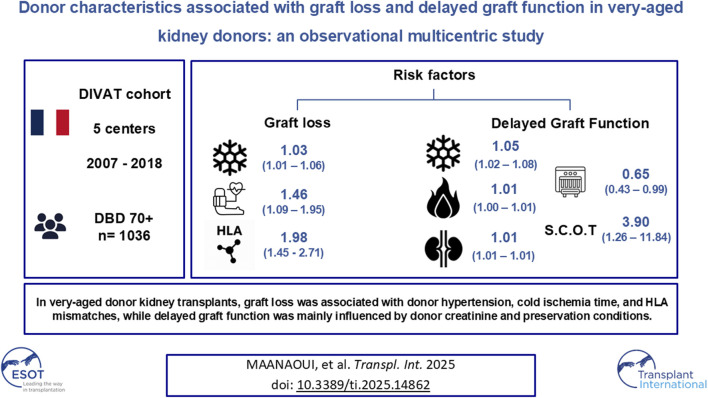

## Introduction

Organs of very-aged kidney donors are a reliable source to increase the pool of organs in a context of shortage [1]. Yet, there are still significant differences between countries. To illustrate, in 2021 6.0% of kidney donors were over 65 years-old in the US, compared to 28.3% in France. The rate of discarded kidneys, which is almost twice as high in the US than in France, partly explains this difference [2]. In order to improve the use of these marginal donors, there is a need to understand which modifiable or non-modifiable risk factors are associated with the risk of graft loss. However, as most allocation programs promote old-for-old strategies [3], the impact of donor aging might be modified by the influence of recipient aging. The objectives of our study are then to identify risk factors of graft loss and delayed graft function in donors aged over 70 years old, independently from recipients’ characteristics.

## Patients and Methods

### Data Source and Ethical Statement

The study protocol adhered to French laws and was performed according to the Declaration of Helsinki and the Declaration of Istanbul. These measures involved approval, pseudonymization, and protective measures in accordance with the local institutional review board and agreement No. 914184 issued by the Commission Nationale de l’informatique et des libertés (the National Commission on Informatics and Liberties, CNIL). This study Data were extracted from the French multicentric, observational and prospective DIVAT cohort (www.divat.fr, ClinicalTrials.gov recording NCT02900040). No organs were procured from prisoners. All recipients gave informed consent to participate in the study. Donor consent requirement was waived in accordance to French policies.

### Study Design

This is a retrospective multicentric study performed on the prospective data collection from five transplant centers from the DIVAT consortium (Lyon, Montpellier, Nice, Nancy and Nantes). All consecutive adult recipients who were transplanted between the 1st of January 2007 and the 31st of December 2018, with a kidney from brain-deceased kidney donors aged were included, whatever the rank of transplantation. Patients transplanted with other organs were non-included. Of note, there was no donation after circulatory death (DCD) donors in the study as the French DCD program excludes donor over 70. The end of follow-up consisted in: the date of the last visit in case of functional graft, the date of return to dialysis, the date of retransplantation, or the date of death.

The first step of the analyses and primary endpoint evaluation involved only transplantation performed from kidney donors over 70. The second step of the analyses, i.e., interaction tests, involved the whole cohort.

The following donor parameters were collected: age, sex, BMI, blood type, HLA antigens, comorbidities (diabetes, hypertension, dyslipidemia), cause of death, hemodynamic data (cardiac arrest, use of pressor amines), renal function (last serum creatinine, last serum urea, and proteinuria) before organ procurement, side of kidney procurement, number of renal arteries. Cold and warm ischemia times as well as the conservation modality (hypothermic perfusion machine (HPM) or static cold storage, type of perfusion or conservation liquid) were also registered.

The following recipient baseline parameters were collected: age, sex, BMI, blood type, transplant center, year of transplant, rank of transplantation, time on dialysis, time on waiting list, type of dialysis, cause of end stage renal disease (ESRD), HLA antigens, HLA sensitization, comorbidities (diabetes, hypertension, cancer, peripheral vascular diseases, urological diseases, smoking).

### Criteria of Judgement

The primary endpoint of our study was death-censored graft survival, graft loss being defined as returning to dialysis or receiving a new pre-emptive retransplant. Our secondary endpoint was the occurrence of delayed graft function (DGF), defined as the need for dialysis, whatever the reason, within the first 7 days.

### Statistical Analysis

Quantitative variables were described by the median and interquartile range and qualitative variables by the number and the percentage. The quantitative variables were compared by the Student’s t-test and the categorical variables by the Chi-square test. The survival curves were obtained using the Kaplan Meier estimator. A multiple imputation method was performed to accommodate missing data on relevant variables. The missing data rate was less than 10% for each imputed variable. Five imputed datasets were constructed and analyses were performed on each of them. The results presented were obtained by combining the results according to the rules of Rubin [4]. The search for donor-related risk factors for graft loss was carried out only in 70+ kidney donors using proportional risk models. First, a Cox model was built with only covariates from the recipient. The variables were selected from the literature and those recognized as risk factors were included in the model, regardless of their significance. Based on the known risk factors for graft loss [5–9], the characteristics of the recipients retained were: year of the transplant, rank of the transplant, age, sex, BMI, waiting time on dialysis and the level of HLA sensitization. The model obtained will be called the “recipient model.” In a second step, each characteristic of the donor was tested separately within the recipient model. The donor variables significant at a threshold of 20% were retained. These were then all integrated into the recipient model and a top-down selection procedure at a 5% threshold was applied only to donor characteristics. This strategy aimed to select donor variables which have a direct causal effect on graft survival, in a way that is not mediated by recipient characteristics. Consequently, donor variables located on the causal pathway between recipient characteristics and the risk of graft loss would not be retained. The log linearity and proportional hazards assumptions were assessed graphically. The study of delayed-graft function was carried out using logistic regression with the same variable selection strategy, meaning the variables were selected from the literature and those recognized as risk factors were included in the model, regardless of their significance. The characteristics of the recipient retained in the literature [10–12] were: year of transplantation, transplant center, age, sex, BMI, level of sensitization, induction therapy, a history of diabetes or peripheral vascular diseases.

Interaction analyses were then performed in the whole cohort, comparing kidney donors aged ≥70 years versus <70 years. Interaction terms were introduced into the Cox proportional hazards models or logistic regression models to assess whether the donor age category modified the assessed associations. The significance of the interaction was evaluated using Wald test on the interaction term. The log linearity assumption was assessed graphically. All analyses were performed with R 3.6.3 software.

## Results

### Baseline Characteristics

Between 2007 and 2018, 5350 kidney transplantations were performed and 1036 patients benefited from a kidney transplant using a graft from a deceased donor over 70 years old. Donors and recipients characteristics are detailed in Table 1. Briefly, median donor and recipients ages were 75 years (72–79) and 69 years (65–73), respectively. Median cold ischemia time was 16.3 h (13.4–19.8), and a hypothermic perfusion machine was used in 47.4% donors. With a median follow-up of 3.96 years (2.01–6.31), 196 deaths and 233 returns to dialysis were observed. Death-censored graft survival probabilities at 1, 3 and 5 years were 88.9% (86.7%–91.1%), 82.7% (79.8%–86.7%) and 77.8% (73.9%–81.7%), respectively (Supplementary Figure S1).

**TABLE 1:** Baseline characteristics of donors aged over 70 and their recipients.

Characteristics	n = 1036
Donor	
Age (years), median (IQR)	75.0 (72.0–79.0)
Death from vascular cause, n (%)	794 (76.6)
Sex (female), n (%)	593 (57.4)
BMI (kg/m^2^), median (IQR)	26.0 (23.1–29.2)
Blood type, n (%)	
A	497 (48.1)
AB	44 (4.3)
B	76 (7.4)
O	416 (40.3)
Diabetes	155 (16.1)
Dyslipidemia	232 (30.7)
Hypertension	610 (61.4)
Recovered cardiac arrest	101 (9.8)
Use of pressor amines	868 (88.0)
Last serum creatinine (μmol/L)	72.0 (56.0–91.0)
Last serum urea (mmol/L)	5.2 (4.0–6.9)
Positive proteinuria	316 (31.5)
Positive hematuria	642 (65.2)
Presence of two arteries or more	158 (15.7)
Recipient	
Age (years), median (IQR)	69.0 (65.0–73.0)
First kidney transplantation, n (%)	892 (86.1)
Sex (female), n (%)	373 (36.0)
BMI (kg/m^2^), median (IQR)	25.5 (23.0–28.4)
Blood type, n (%)	
A	502 (48.7)
AB	50 (4.9)
B	80 (7.8)
O	399 (38.7)
Type of dialysis	
Hemodialysis, n (%)	791 (76.6)
Peritoneal dialysis, n (%)	112 (10.8)
Preemptive transplantation, n (%)	130 (12.6)
Cause of ESKD	
Glomerulonephritis, n (%)	192 (18.5)
Vascular nephropathy, n (%)	174 (16.8)
Undetermined, n (%)	167 (16.1)
Diabetes, n (%)	160 (15.4)
Tubulo-interstitial nephritis, n (%)	343 (33.1)
Comorbidities, n (%)	
Diabetes	295 (28.5)
Dyslipidemia	532 (51.4)
Hypertension	867 (83.7)
Cancer	239 (23.1)
Smoking	410 (60.9)
Peripheral vascular diseases	561 (54.2)
Waiting time on dialysis (years), median (IQR)	2.1 (1.0–3.7)
HLA sensitization class I, n (%)	286 (33.0)
HLA sensitization class II, n (%)	253 (29.6)
Transplantation	
Cold ischemia time (h), median (IQR)	16.3 (13.4–19.8)
Warm ischemia time (min), median (IQR)	40.0 (31.0–50.0)
Hypothermic perfusion machine, n (%)	470 (47.4)
Preservation solution, n (%)	
UW	258 (25.6)
S.C.O.T	19 (1.90)
CELSIOR	233 (23.4)
IGL	302 (30.3)
Other	187 (18.8)
ABDR mismatches >4, n (%)	234 (22.9)
Induction therapy	
Thymoglobulin	411 (40.1)
Anti-IL2 receptor	591 (57.6)
Other	24 (2.3)

### Risk Factors for Graft Loss

We first performed a cox-regression model including only recipient variables, resulting in a fully adjusted model on recipient variables, defined as “recipient model” (not shown). Donor variables significantly associated with the probability of death-censored graft survival in multivariate analyses were then: a history of hypertension in the donor (HR 1.46 (1.09–1.95)) and cold ischemia (HR per hour 1.03 (1.01–1.06)) (See Table 2).

**TABLE 2:** Donor-related multivariate Cox regression model for the risk of death-censored graft loss.

Variables	Death-censored graft loss	
	Multivariate HR [95%CI]	*p*-value
Hypertension	1.46 (1.09–1.95)	0.01
Cold ischemia time (per hour)	1.03 (1.01–1.06)	0.01
ABDR mismatches (>4 vs. ≤4)	1.35 (0.99–1.84)	0.053

Every donor variables were included in a model adjusted on recipient variables, i.e.,: year of transplantation, rank of transplantation, recipient age, recipient sex, recipient BMI, waiting time on dialysis and class I and II HLA sensitization.

### Risk Factors for Delayed Graft Function

Within the whole cohort, 57 recipients lost their graft or died before day 7 post-KTx, and 2 had missing data. Among the 977 remaining recipients, 283 (29.0%) experienced a delayed graft function. Baseline characteristics of recipients experiencing or not DGF are depicted Table 3. As for the graft survival analysis, we first built a fully adjusted logistic regression model on recipient variables (Table 4). Donor last serum creatinine was significantly associated with the risk of delayed graft function (HR 1.01 (1.01–1.01) per creatinine point in μmol/L), as well as warm (HR 1.01 (1.0–1.01) per minutes) and cold ischemia times (HR 1.05 (1.02–1.08) per hour). Concerning the condition of organ preservation, S.C.O.T. preservation solution was associated with the risk of DGF (HR 3.90 (1.26–11.84)). Conversely, the use of a hypothermic perfusion machine was a protective factor for the occurrence of DGF (HR 0.65 (0.43–0.99)).

**TABLE 3:** Baseline characteristics of donors aged over 70 and their recipients, with or without delayed-graft function.

Characteristics	No DGF (n = 694)	DGF (n = 283)	p-value
Donor			
Age (years), median (IQR)	75.0 (72.0–79.0)	75.0 (72.0–78.0)	0.10
Death from vascular cause, n (%)	530 (76.4)	217 (76.7)	0.98
Sex (female), n (%)	272 (33.2)	142 (50.2)	<0.01
BMI (kg/m^2^), median (IQR)	25.8 (22.9–29.1)	26.1 (23.5–29.2)	0.26
Blood type, n (%)			0.11
A	346 (50.1)	123 (43.5)	
AB	33 (4.8)	9 (3.2)	
B	51 (7.4)	23 (8.1)	
O	261 (37.8)	128 (45.2)	
Diabetes	99 (12.3)	45 (12.2)	0.52
Dyslipidemia	147 (30.0)	69 (32.7)	0.37
Hypertension	388 (58.2)	182 (66.2)	0.04
Recovered cardiac arrest	67 (9.7)	31 (10.1)	0.62
Use of pressor amines	575 (87.4)	244 (89.7)	0.38
Last serum creatinine	71.0 (56.0–90.0)	76.0 (58.0–96.0)	0.02
Last serum urea	5.1 (3.9–6.8)	5.51 (4.3–7.2)	<0.01
Positive proteinuria	220 (32.3)	79.0 (28.6)	0.69
Positive hematuria	435 (66.2)	175 (64.6)	0.69
Presence of two arteries or more	108 (16.1)	41 (14.8)	0.68
Recipient			
Age (years), median (IQR)	69.0 (64.0–73.0)	69.0 (65.0–74.0)	0.64
First kidney transplantation, n (%)	611 (88.0)	235 (83.0)	0.05
Sex (female), n (%)	252 (36.3)	93 (32.9)	0.34
BMI (kg/m^2^), median (IQR)	25.2 (22.7–27.9)	26.5 (23.5–29.4)	<0.01
Blood type, n (%)			0.14
A	350 (50.8)	122 (43.1)	
AB	34 (4.9)	13 (4.6)	
B	54 (7.8)	24 (8.5)	
O	251 (36.4)	124 (43.8)	
Type of dialysis			<0.01
Hemodialysis, n (%)	473 (68.3)	270 (95.4)	
Peritoneal dialysis, n (%)	94 (13.6)	10 (3.5)	
Preemptive transplantation, n (%)	125 (18.1)	3 (1.1)	
Cause of ESKD			0.13
Glomerulonephritis, n (%)	131 (18.9)	53 (18.7)	
Vascular nephropathy, n (%)	117 (16.9)	55 (19.4)	
Undetermined, n (%)	120 (17.3)	34 (12.0)	
Diabetes, n (%)	94 (13.5)	51 (18.0)	
Tubulo-interstitial nephritis, n (%)	131 (18.9)	53 (18.7)	
Comorbidities, n (%)			
Diabetes	179 (25.8)	96 (33.9)	0.01
Dyslipidemia	351 (50.6)	156 (55.1)	0.22
Hypertension	580 (83.6)	156 (55.1)	0.22
Cancer	160 (23.1)	64 (22.6)	0.95
Smoking	277 (62.0)	112 (59.3)	0.58
Peripheral vascular diseases	347 (50.0)	181 (64.00)	<0.01
Waiting time on dialysis (years), median (IQR)	1.8 (0.6–3.4)	2.7 (1.6–4.3)	<0.01
HLA sensitization class I, n (%)	196 (32.7)	73 (32.2)	0.96
HLA sensitization class II, n (%)	164 (27.7)	74 (33.2)	0.14
Transplantation			
Cold ischemia time (h), median (IQR)	15.8 (13.15–19.0)	17.4 (13.9–21.0)	<0.01
Warm ischemia time (min), median (IQR)	40.0 (31.0–50.0)	42.50 (34.0–55.0)	<0.01
Hypothermic perfusion machine, n (%)	324 (49.2)	119 (43.3)	0.12
Preservation solution, n (%)			<0.01
UW	131 (24.4)	60 (34.6)	
S.C.O.T	6 (0.9)	11 (4.0)	
CELSIOR	149 (22.5)	74 (26.7)	
IGL	224 (33.8)	59 (21.3)	
Other	122 (18.4)	37 (13.4)	
ABDR mismatches >4, n (%)	155 (22.5)	65 (23.6)	0.79
Induction therapy			0.51
Thymoglobulin	277 (40.3)	117 (41.3)	
Anti-IL2 receptor	398 (57.9)	158 (55.8)	
Other	12 (1.8)	8 (2.8)	

**TABLE 4:** Donor-related logistic regression model for the risk of delayed-graft function.

Variables	Delayed graft function	
	Multivariate OR [95% CI]	*p*-value
Donor serum creatinine (per μmol/L)	1.01 (1.01–1.01)	<0.01
Cold ischemia time (per hour)	1.05 (1.02–1.08)	<0.01
Warm ischemia time (per hour)	1.01 (1.00–1.01)	0.04
S.C.O.T preservation solution	3.86 (1.26–11.86)	0.02
Perfusion machine	0.65 (0.43–0.99)	0.04

Every donor variables were included in a model adjusted on recipient variables, i.e.,: transplantation center, year of transplantation, rank of transplantation, recipient age, recipient sex, recipient BMI, waiting time on dialysis and class I and II HLA sensitization, induction therapy, recipient diabetes and recipient vascular diseases.

To end with, we evaluated the effect of DGF on the risk of death-censored graft survival. As described above, we first built a fully adjusted “recipient model,” testing then one by one each donor variable, as well as delayed graft function as a covariate of interest. Baseline was defined as day 7 post-KTx. DGF was significantly associated with the risk of death-censored graft failure with a HR of 1.94 (1.38–2.73). The other variable that remained significant was the number of ABDR mismatches with a HR of 1.50 (1.06–2.13), if there were more than 4 mismatches (Supplementary Table S1).

### Evaluation of Age-Specificity Impact of Risk Factors of Death-Censored Graft Loss or Delayed Graft Function

We tested then the interaction between age categories (i.e. 70+ or 70−) and the identified risk factors of death-censored graft survival or delayed graft function (Table 5). Considering death-censored graft survival, hypertension was age-specific with an increased risk in 70+ kidney donors (HR 2.14 (1.75–2.62), p < 0.001) as well as the number of ABDR mismatches (HR 1.98 (1.45–2.71), p < 0.001). We did not find any significant age-specific impact of cold ischemia time, even though there seemed to be a trend (70+: HR 1.04 (1.03–1.05) versus 70− HR 1.00 (0.99–1.01), p = 0.18). Considering delayed-graft function, we did not find any significant interaction between age categories and the identified risk factors for DGF.

**TABLE 5:** Interaction tests between risk factors of death-censored graft loss or delayed graft function and age categories.

Variables	Multivariate HR (95%CI)	*p-value*
Death-censored graft loss		
Hypertension		
70+	2.14 (1.75–2.62)	<0.001
70-	1	
Cold ischemia time (per hour)		
70+	1.04 (1.03–1.05)	0.18
70-	1.00 (0.99–1.01)	
ABDR mismatches (>4 vs. ≤4)		
70+	1.98 (1.45–2.71)	<0.001
70-	1	
Delayed graft function		
Donor serum creatinine (per μmol/L)		
70+	1.01 (1.006–1.011)	0.35
70-	1.007 (1.006–1.008)	
Cold ischemia time (per hour)		
70+	1.05 (1.03–1.06)	0.36
70-	1.04 (1.03–1.05)	
Warm ischemia time (per hour)		
70+	1.011 (1.006–1.016)	0.73
70-	1.009 (1.004–1.014)	
S.C.O.T preservation solution		
70+	2.18 (0.73–6.76)	0.18
70-	1	

## Discussion

In this study, we provide evidence that donor hypertension and cold ischemia time are associated with graft loss in DBD very-aged kidney donors. Risk factors of delayed graft function were mainly related to the conditions of organ procurement and processing, i.e., cold and warm ischemia times, cold-storage compared to the use of hypothermic perfusion machine, and finally the use of SCOT preservation solution. The last donor serum creatinine was also associated with delayed graft function. These results are in favor of a global call to enforce donor protective strategies.

Considering the shortage of organs there is a need to identify available sources easy to scale. Discrepancies between allocation systems regarding the percentage of very-old donors point out these donors as a potential target. Yet, raw survivals of these marginal donors may look insufficient in a utilitarian system compared to younger donors, which explain why these donors are more often discarded [13]. However, the population is aging, and transplant systems face an increasingly older recipient population registered on the waiting list [14, 15]. “Old-for-old” organ allocation have been proposed for these older recipients, in which they get attributed older donors [16]. As there is a global collinearity between donor and recipient aging, one may argue that these raw poor survivals might reflect recipient characteristics much more than the donor ones. There is thus a need to identify donor-related modifiable or non-modifiable risk factors associated with poor outcomes, i.e., graft loss or delayed graft function, independently from recipient-related variables, in order to anticipate preventive strategies and facilitate the use of these marginal organs.

As far as graft survival is concerned, we finally identified very few donor-related variables. We performed two regression models, in which death-censored graft survival was the outcome, including or not delayed graft function as a covariate. When DGF was not included, donor hypertension and cold ischemia time were significantly associated with graft loss and A/B/DR mismatches almost significant. After adjustment on DGF, both donor hypertension and cold ischemia time HR decreased drastically and were not significant anymore. On the contrary, A/B/DR mismatches became significantly associated with graft loss. Of note, only donor hypertension and A/B/DR mismatches were age-specific. Donor hypertension is a non-modifiable risk factor reputedly described as associated with graft loss, as *per se* as part of the definition of expanded-criteria donor. However, we saw a notable decrease of the risk associated with donor hypertension after adjustment with DGF, from a HR of 1.46 (1.09–1.945) to 1.17 (0.85–1.63). As far as we know, no studies assessed the interrelationship between hypertension, delayed graft function and graft loss, even though donor hypertension has been shown to be associated with the risk of DGF [17, 18]. Thus, and based on our findings, delayed graft function might stand between donor hypertension and graft loss in the causal pathway, as suggested by Debout et al. [19] and may partly explained the risk associated with donor hypertension on long-term outcomes. In parallel, the same analysis can be drawn to cold ischemia time, which is a modifiable risk factor. It is also well-known as being associated with graft survival [19, 20] notably in donor aged over 70 [21]. In our study, the HR went from 1.03 per added hour (1.01–1.06) before adjustment on DGF, to 1.02 (0.99–1.05) after adjustment, highlighting the assumption of DGF being in the causal pathway between cold ischemia time and graft loss. To mitigate the risk induced by cold ischemia time, several strategies may be considered. For instance, the use of virtual crossmatch compared to lymphocytoxicity crossmatch when an elderly kidney donor is allocated may be a simple way to reduce cold ischemia time, as studies found a 1.5–3 h reduction of CIT when virtual crossmatch was implemented [22, 23]. Kidney allocation strategies could also be reconsidered, with specific allocation pathways dedicated to elderly donors. For instance, the Eurotransplant Senior Program, implemented in 1999, was designed to facilitate regional allocation of kidneys from older donors to elderly recipients, thereby reducing cold ischemia time. Following its implementation, data demonstrated an approximate two-hour reduction in cold ischemia duration [24, 25].

Finally, the only variable that became significant after adjustment on DGF was HLA A/B/DR mismatches, which is a modifiable factor. This is consistent with previous findings on the risk of graft loss associated with HLA mismatches [16, 21] in very old donors and recipients. This pleads for HLA-based matching algorithm for the elderly, such as the example of the Eurotransplant HLA-DR matching experience described by de Fijter et al. In the Eurotransplant senior program, matching for HLA-DR antigens was associated with a significantly lower risk of kidney graft loss at 5 years, with a HR of 0.73 (0.53–0.99) post-transplant. Another example comes from the United Kingdom, with the recent implementation of the D4–R4 allocation scheme. In this system, both donors and recipients are stratified according to their estimated risk of graft failure, with higher-risk donors (D1 to D4, D4 holding the higher risk) preferentially allocated to higher-risk recipients (R1 to R4, R4 holding the higher risk) [26]. Notably, donors over 70 years of age classified in the D4 category are subject to a specific allocation rule: their kidneys are offered as dual transplants to the center with the largest number of listed patients (as defined by the National Kidney Offering Scheme), although that center retains the option to transplant one or both kidneys into any locally listed recipient [27].

Considering delayed-graft function as an outcome, we found several variables that are known to influence the risk of DGF, i.e., cold and warm ischemia times and the last donor serum creatinine [18]. Prolonged cold and warm ischemia times have been shown to be independent predictors of DGF, especially in ECD donors [28, 29]. These results are in favor of strategies targeting to reduce cold and warm ischemia times, both modifiable factors. To illustrate, factors that may influence warm ischemia time involve the vascular complexity, e.g., the number of renal arteries [30, 31]. Per se, it does represent a contraindication of kidney transplantation, however this might be considered at the time of implantation, with, for example, a facilitated access to the operation room and the presence of a trained surgeon.

Considering the effect of the last donor serum creatinine on the risk of DGF, even though consistent with the literature, we acknowledge that this study was not performed to evaluate the subtle impact of donor renal function on post-transplantation outcomes. Indeed, the DIVAT cohort does not include donor longitudinal serum creatinine values before organ procurement, thus we could not evaluate the impact of donor AKI, and we previously showed that both donor AKI or peak serum creatinine values could have an impact on post-transplantation renal outcomes, especially in the elderly [32, 33]. Yet, last donor serum creatinine is a simple tool to implement in clinical practice, even though non-modifiable *per se*, in order to anticipate preventive strategies against DGF.

Considering preventive therapeutics against DGF, peri-transplant optimization should be at the heart of these elderly donors’ management. More than 50% of kidneys in our cohort did not benefit from hypothermic perfusion machine, even though randomized clinical trials showed a significant benefit [34]. Guidelines should then recommend the systematic use of hypothermic perfusion machine for elderly donors when available. If unavailable, the type of conservative solution may be discussed. Interestingly, we found a deleterious impact of the S.C.O.T preservation solution, which has been reported before in other large-scale studies [35]. Thus, the avoidance of S.C.O.T may be considered, especially in France, when elderly donors are concerned. Innovative strategies, such as the use of normothermic perfusion machine [36] or hypothermia [37] in deceased donors might be discussed also, yet for now none of them found a significant benefits compared to conventional therapies. The choice of peritransplant fluids might be a simpler strategy compared to those which have been discussed earlier. Collins et al. [38] recently found that the use of balanced crystalloids versus saline significantly reduced the rate of DGF from 40% to 30% when transplanting kidneys from deceased donors. This could advocate to systematically consider balanced crystalloids in case of kidney transplantation from elderly donors.

Our work has some limits. These results are based on a French prospective cohort, which may not globalize to other allocation system and populations. As ethnicity cannot be analysed in France, we cannot provide any elements regarding the impact of ethnicity in our models. Furthermore, there is a risk of selection bias, as donor selection is dependent on self-practices, and extreme characteristics may be systematically avoided. This study does not provide evidence to accept or discard any subpopulation of donors, as the objectives was to only determine risk factors associated with impaired outcomes, and did not involve any control group. Finally, while our study focused on assessing the isolated impact of donor factors by holding recipient variables constant, an important question remains as to whether the effect of very old donor kidneys could be modified by recipient age. One could hypothesize that younger recipients might mitigate some of the risks associated with donor aging. Our analysis was not designed to formally evaluate such interactions, but this represents a valuable avenue for future research to better understand the interplay between donor and recipient aging in kidney transplantation.

Ultimately, we found several modifiable or non-modifiable risk factors of graft loss or delayed graft function, which was consistent with what has been described in the literature. These provide incentives to further implement strategies targeting this specific population of very-old kidney donors.

## Data Availability

The raw data supporting the conclusions of this article will be made available by the authors, without undue reservation.

## Group Members of DIVAT Consortium

Nantes: Gilles Blancho, Julien Branchereau, Diego Cantarovich, Agnès Chapelet, Jacques Dantal, Clément Deltombe, Lucile Figueres, Claire Garandeau, Magali Giral, Caroline Gourraud-Vercel, Clarisse Kerleau, Thibault Letellier, Christophe Masset, Aurélie Meurette, Simon Ville, Christine Kandel, Anne Moreau, Karine Renaudin, Alexandre Walencik, Laetitia Anquetil; Lyon: Lionel Badet, Xavier Charmetant, Fanny Buron, Ricardo Codas, Sameh Daoud, Valérie Dubois, Alice Koenig, Emmanuel Morelon, Luc Chauvelot, Manon Guézennec, Maud Rabeyrin, Anne-Claire Lukaszewicz, Olivier Thaunat; Montpellier: Nicolas Abdo, Sylvie Delmas, Moglie Le Quintrec, Vincent Pernin, Hélène Perrochia, Jean-Emmanuel Serre, Ilan Szwarc; Nice: Laetitia Albano, Damien Ambrosetti, Nadia Ben Hassen, Mathilde Blois, Marion Cremoni, Matthieu Durand, Patricia Goldis, Clément Gosset, Fatimaezzahra Karimi, Antoine Sicard, Giorgo Toni; Nancy: Alice Aarnink, Asma Alla, Pascal Eschwege, Luc Frimat, Sophie Girerd, Jacques Hubert, Raphaël Kormann, Marc Ladriere, François Lagrange, Emmanuelle Laurain, Pierre Lecoanet, Jean-Louis Lemelle; Anthony Mannuguerra, Charles Mazeaud, Michael Peres.
