# Supplementary material for: Donor Characteristics Associated With Graft Loss and Delayed Graft Function in Very-Aged Kidney Donors: An Observational Multicentric Study
Source: Transpl Int. 2025 Nov 18;38:14862. doi: 10.3389/ti.2025.14862 (PMC12669066; doi:10.3389/ti.2025.14862)
Supplement: Supplementary file 1 [file DataSheet1.pdf]

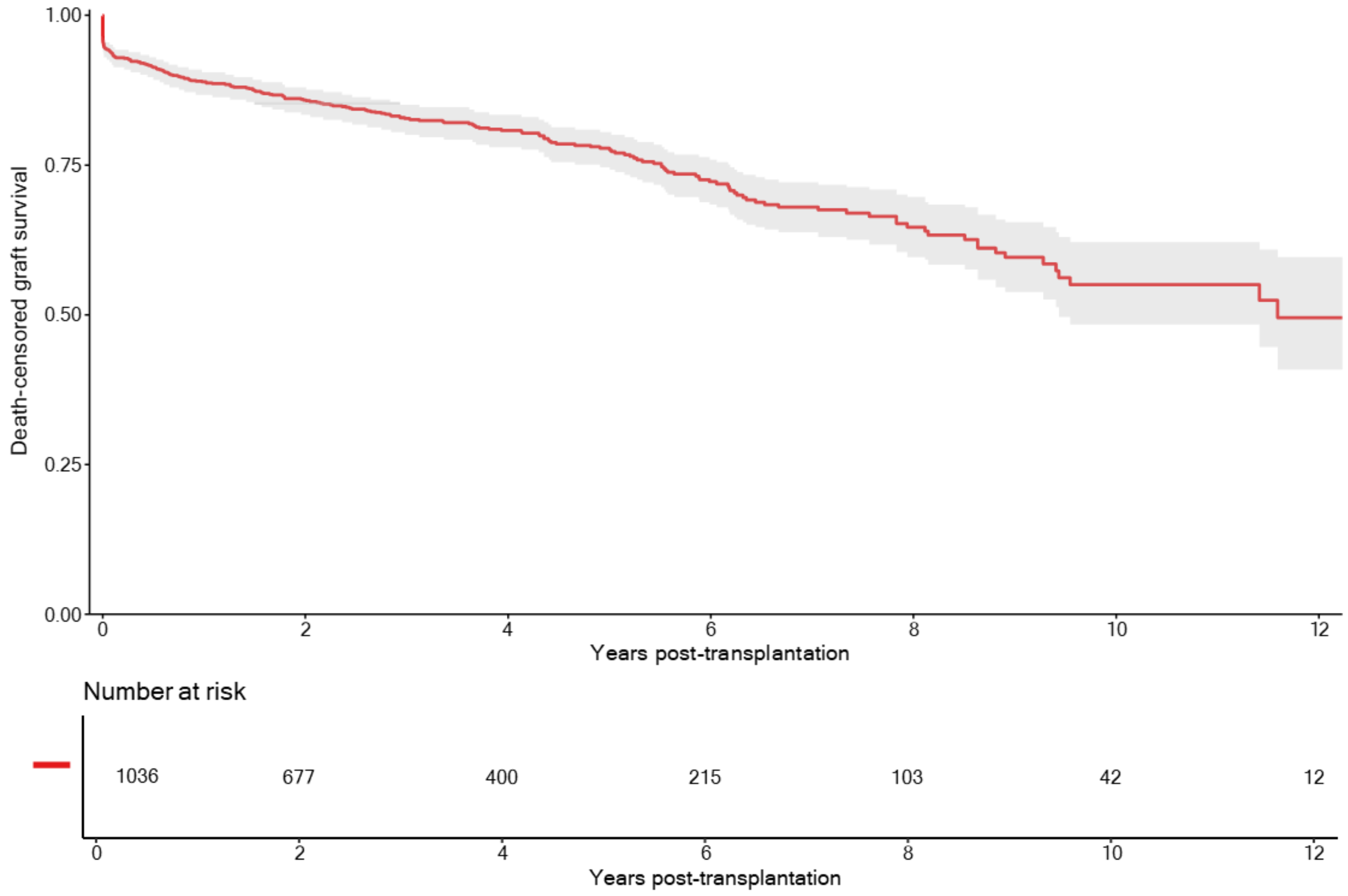

Supplemental Figure 1. Kaplan-Meier death-censored graft survival curve in patients transplanted from a very-aged kidney donor over 70 years-old.

Supplemental Table 1. Donor-related multivariate Cox regression model for the risk of death-censored graft loss, including delayed-graft function as a covariate.

	Death-censored graft loss	
	Multivariate HR [95%CI]	*p*-value
Delayed graft function	1.94 (1.38 - 2.73)	< 0.01
Hypertension	1.17 (0.85 - 1.63)	0.34
Cold ischemia time (per hour)	1.02 (0.99 - 1.05)	0.16
ABDR mismatches (>4 vs ≤4)	1.50 (1.06 - 2.13)	0.02

Every donor variables were included in a model adjusted on recipient variables, i.e: year of transplantation, rank of transplantation, recipient age, recipient sex, recipient BMI, waiting time on dialysis and class I and II HLA sensitization.
